# Supplementary material for: Improvement of Colonic Immune Function with Soy Isoflavones in High-Fat Diet-Induced Obese Rats
Source: Molecules. 2019 Mar 22;24(6):1139. doi: 10.3390/molecules24061139 (PMC6470843; doi:10.3390/molecules24061139)
Supplement: Supplementary file 1 [file molecules-24-01139-s001.pdf]

Supplementary material: the following are supplementary materials, Figure S1: The content of sIgA and 8-OHDG, Figure S2: Bacterial taxonomic analysis of intestinal bacterium from each rat in the phylum, Figure S3. Relative abundance of several microbiota in the genus.

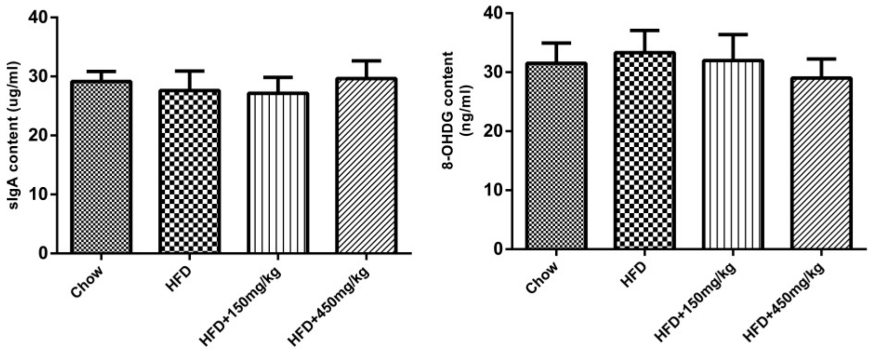

Figure S1. (**a**) The content of sIgA in colonic mucosa detected by ELISA; (**b**) The content of 8-OHDG in colonic mucosa detected by ELISA.

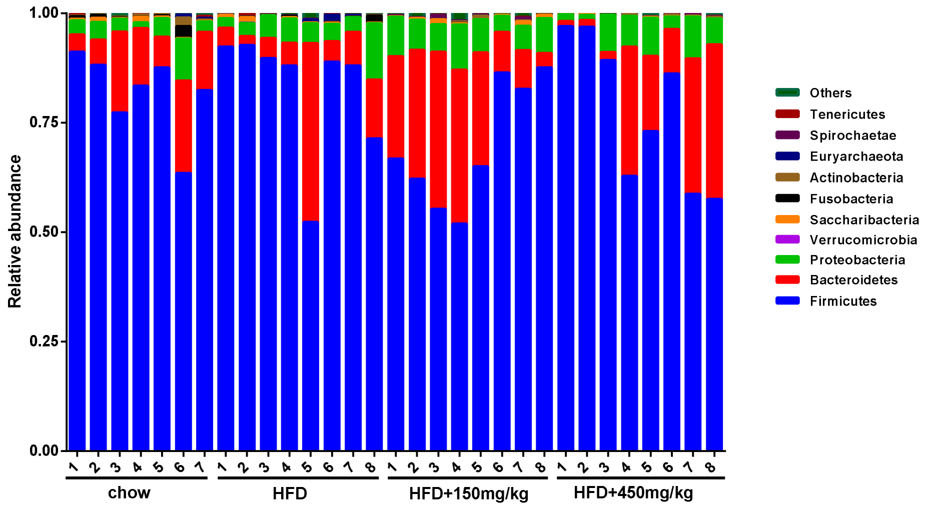

Figure S2. Bacterial taxonomic analysis of intestinal bacterium from each rat in the phylum.

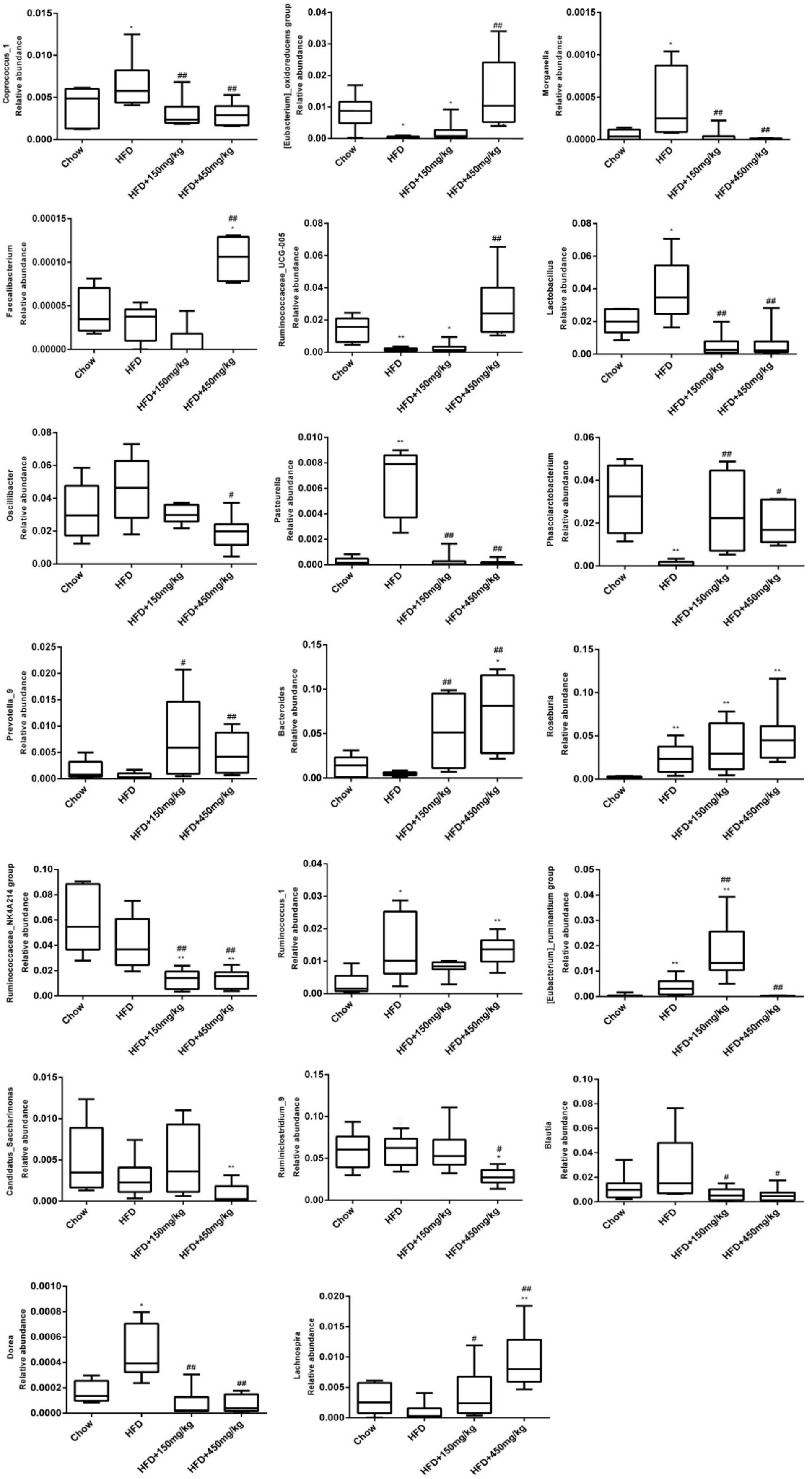
Coprococcus_1
[Eubacterium]_oxidoreducens group
Morganella
Faecalibacterium
Ruminococcaceae_UCG-005
Lactobacillus
Oscillibacter
Pasteurella
Phascolarctobacterium
Prevotella_9
Bacteroides
Roseburia
Ruminococcaceae_NK4A214 group
Ruminococcus_1
[Eubacterium]_ruminantium group
Candidatus_Saccharimonas
Ruminiclostridium_9
Blautia
Dorea
Lachnospira
Relative abundance
Chow
HFD
HFD+150mg/kg
HFD+450mg/kg

Figure S3. Relative abundance of several microbiota in the genus (those with significant difference among groups). P values were indicated as: *P< 0.05, **P< 0.005(* indicate versus the chow group). $^{\#}$P< 0.05, $^{\#\#}$P< 0.005($^{\#}$ indicate versus the HFD group).
